# Atherosclerotic plaque features relevant to rupture-risk detected by clinical photon-counting CT *ex vivo*: a proof-of-concept study

**DOI:** 10.1186/s41747-023-00410-4

**Published:** 2024-01-30

**Authors:** Annelie Shami, Jiangming Sun, Chrysostomi Gialeli, Hanna Markstad, Andreas Edsfeldt, Marie-Louise Aurumskjöld, Isabel Gonçalves

**Affiliations:** 1 Department of Clinical Sciences Malmö, Lund University, Clinical Research Center, Jan Waldenströms Gata 35, CRC 91:12, 214 28 Malmö, Sweden; 2 Department of Medical Imaging and Physiology, Skåne University Hospital, Lund/Malmö, Sweden; 3 Department of Cardiology, Malmö, Skåne University Hospital, Lund University, Lund, Sweden; 4 Wallenberg Centre for Molecular Medicine, Lund University, Lund, Sweden; 5 Department of Clinical Sciences Malmö, Medical Radiation Physics, Skåne University Hospital, Lund University, 205 02 Malmö, Sweden; 6 Department of Hematology, Oncology and Radiation Physics, Radiation Physics, Skåne University Hospital, Lund, Sweden

**Keywords:** Atherosclerosis, Carotid arteries, Carotid stenosis, Plaque (atherosclerotic), Tomography (x-ray computed)

## Abstract

**Background:**

To identify subjects with rupture-prone atherosclerotic plaques before thrombotic events occur is an unmet clinical need. Thus, this proof-of-concept study aims to determine which rupture-prone plaque features can be detected using clinically available photon-counting computed tomography (PCCT).

**Methods:**

In this retrospective study, advanced atherosclerotic plaques (*ex vivo*, paraffin-embedded) from the Carotid Plaque Imaging Project were scanned by PCCT with reconstructed energy levels (45, 70, 120, 190 keV). Density in HU was measured in 97 regions of interest (ROIs) representing rupture-prone plaque features as demonstrated by histopathology (thrombus, lipid core, necrosis, fibrosis, intraplaque haemorrhage, calcium). The relationship between HU and energy was then assessed using a mixed-effects model for each plaque feature.

**Results:**

Plaques from five men (age 79 ± 8 [mean ± standard deviation]) were included in the study. Comparing differences in coefficients (*b*_1diff_) of matched ROIs on plaque images obtained by PCCT and histology confirmed that calcium was distinguishable from all other analysed features. Of greater novelty, additional rupture-prone plaque features proved discernible from each other, particularly when comparing haemorrhage with fibrous cap (*p* = 0.017), lipids (*p* = 0.003) and necrosis (*p* = 0.004) and thrombus compared to fibrosis (*p* = 0.048), fibrous cap (*p* = 0.028), lipids (*p* = 0.015) and necrosis (*p* = 0.017).

**Conclusions:**

Clinically available PCCT detects not only calcification, but also other rupture-prone features of human carotid plaques *ex vivo*.

**Relevance statement:**

Improved atherosclerotic plaque characterisation by photon-counting CT provides the ability to distinguish not only calcium, but also rupture-prone plaque features such as haemorrhage and thrombus. This may potentially improve monitoring and risk stratification of atherosclerotic patients in order to prevent strokes.

**Key points:**

• CT of atherosclerotic plaques mainly detects calcium.

• Many components, such as intra-plaque haemorrhage and lipids, determine increased plaque rupture risk.

• *Ex vivo* carotid plaque photon-counting CT distinguishes haemorrhage and thrombus.

• Improved plaque photon-counting CT evaluation may refine risk stratification accuracy to prevent strokes.

**Graphical Abstract:**

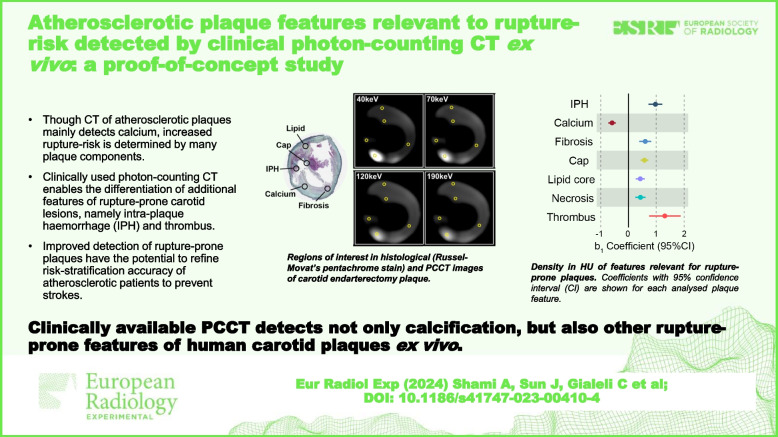

**Supplementary Information:**

The online version contains supplementary material available at 10.1186/s41747-023-00410-4.

## Background

Thrombus formation due to the rupture of an atherosclerotic plaque is the main underlying cause of cardiovascular events, including stroke and myocardial infarction [1]: the world’s largest causes of death and disability [2]. Identifying patients with plaques at risk of rupture, otherwise known as vulnerable plaques, would therefore be of great clinical value. Most rupture-prone plaques are histologically characterised by a large lipid and/or necrotic core, often with intraplaque haemorrhage (IPH) and covered by a thin fibrous cap rich in inflammatory cells [3].

Computed tomography (CT) is a well-known method for non-invasive vascular imaging that now has an important role in clinical guidelines [4, 5]. Photon counting CT (PCCT) is the latest CT technology that involves the acquisition of spectral information with a high resolution, reduced electronic noise and increased contrast-to-noise [6]. This leads to additional image data, finer image quality and lower radiation dose.

While traditional CT is particularly praised for visualisation of calcium content in plaques [7, 8], an increased plaque rupture risk is, in reality, determined by many components other than calcium, such as IPH and lipid content [3, 9]. Whether novel PCCT has the potential to detect these rupture-relevant plaque features beyond calcification has not been explored in human atherosclerotic plaques.

Deepened knowledge of the atherosclerotic plaque composition using PCCT would prove a significant step toward determining the risk of plaque rupture. It would thus improve diagnosis and risk stratification and allow monitoring of preventive or therapeutic interventions. In this study, we have assessed which histological characteristics of rupture-prone plaques can be detected using PCCT.

## Methods

Methods are described in detail in Additional file 1.

### Study cohort: the Carotid Plaque Imaging Project

The study was approved by the Swedish Ethical Review Authority and conforms to the principles of the Declaration of Helsinki. All study subjects gave written informed consent. This is a cross-sectional, observational retrospective study of advanced human carotid atherosclerotic plaques selected among specimens from symptomatic patients enrolled in the prospective Carotid Plaque Imaging Project cohort (collected from May 2019 to June 2020). Datasets analysed/generated during this study and containing deidentified participant data are subject to limitations specific to the ethical permit, as well as general data protection regulation (GDPR, (EU) 2016/679). Because of the sensitive nature of the data collected for this study, requests to access the dataset from qualified researchers trained in human subject confidentiality protocols may be sent to the corresponding author.

Five carotid plaques were randomly chosen from donations of patients with carotid stenosis undergoing endarterectomy at the Vascular Department of Skåne University Hospital, Malmö, Sweden (Table 1). Inclusion criteria were as follows: age over 18 years, eligibility for carotid endarterectomy due to advanced atherosclerosis and ability to provide informed consent. Five samples (from patients of the same sex) were studied as proof of concept, as no power calculation would have been possible in advance.

**Table 1:** Clinical characteristics of the included patients. Continuous measurements are shown as mean with standard deviation

Clinical characteristics	Included subjects (*n* = 5)
Sex (male, % male)	5/5 (100%)
Age (years)	79.0 ± 8.0
Body mass index	25.2 ± 2.4
Active smoker	1/5 (20%)
Fasting lipoproteins (mmol/L)	
Total cholesterol	4.3 ± 1.4
Triglycerides	1.3 ± 0.5
Low-density lipoprotein	2.6 ± 1.4
High-density lipoprotein	1.4 ± 0.3
HbA1c (mmol/mol)	35.8 ± 2.4
Preoperative symptoms	5/5 (100%)
Degree of stenosis (%)	67 ± 9.0
Diabetes	0/5 (0%)
Hypertension	4/5 (80%)
Statin use	2/5 (40%)

Categorical variables are shown as ratios (yes/total subjects, if not otherwise specified) and percentages (of ‘yes’, if not otherwise specified). Continuous variables are given as mean ± standard deviation. *HbA1c* Hemoglobin A1c

### PCCT and histopathology

The specimens were scanned on the first whole body, dual-source PCCT for clinical use (Naeotom Alpha, Siemens Healthineers, Erlangen, Germany) using software Syngo CT VA40A. Two different scan protocols were used, first with a high radiation dose and high resolution, using Sn140kVp and 245 effective mAs, and second with a clinical routine protocol for neck vascular 120 kVp and 100 effective mAs. The reconstructions were set at 45, 70, 90, and 180 keV. Image reconstruction was performed using quantum iterative reconstruction algorithm level 3 and reconstruction kernel Bv44. The field of view was 350 mm with an image matrix size of 1,024 × 1,024. For high-resolution images, the slice thickness was set to 0.4 mm and for the clinical scan 1 mm. All images were evaluated on a clinical Picture Archiving and Communication System station (Barco, Kortrijk, Belgium).

### Tissue processing and comparison

Following PCCT scanning, the plaques were cut cross-sectionally, divided into 3 or 4 portions (depending on plaque size), identified by distance measurements (with a ruler in mm), sectioned on a microtome (5 μm), decalcified (in 14% ethylenediaminetetraacetic acid for 72 h) and visualised with histological stains (von Kossa, Russel-Movat’s pentachrome, Glycophorin A, oxidised low-density lipoprotein).

Based on recorded measurements of plaque length and location of the bifurcation (obtained through each PCCT scan as well as during histological sectioning), locations of histological Sects. 4 μm were matched with PCCT image slices. Plaque and image alignment was validated by an imaging cardiologist with more than 20 years of experience interpreting histopathological plaque images and 10 years of experience interpreting CT, who manually reviewed each match by comparing topological landmarks of the plaque, such as lumen size/shape and macro-calcifications.

Regions of interest (ROIs) were determined on histologically visualised plaque sections representing the plaque features calcium, IPH, thrombus, fibrous cap, intraplaque fibrosis (not in the cap), lipid core, and necrotic core (3 to 11 ROIs per section, but never more than one ROI per plaque feature). In total, plaques from five patients (17 plaque portions) were analysed, resulting in 68 histologically stained sections, and 97 ROIs. The number of ROIs per plaque was 14, 14, 15, 29, and 25, respectively, with the number of ROIs depending on plaque size. ROIs were marked on corresponding locations in PCCT images by a blinded medical physicist. Then, HU were measured for each ROI according to the findings in histopathology for four different stains. To ensure correct plaque orientation, ROI was also placed at the lumen.

### Statistical analysis

The relationship between HU and energy was assessed using a mixed-effects model for each respective plaque feature (calcium, fibrosis [excluding the cap], necrosis, lipid core, fibrous cap, intraplaque haemorrhage, and thrombus, Eq. 1) where energy (with levels of 45, 70, 120, and 190 keV) was the independent variable and HU the dependent variable. The mixed-effects model was applied as shown in the Eq. 1 (see below), where the *b*_1_ coefficient represented energy effect (fixed effect). We assumed heterogeneous effects across ROIs (random effect) in the associations between energy and HU for multiple ROIs (random effect), whereas ROIs from the same plaque might be less independent. Prior to analysis, energy was log-transformed to meet the linearity assumption and HU were standardised to make the *b*_1_ coefficients from various plaque features comparable. The *p*-values from the Wald test were reported for the *b*_1_ coefficient against the null hypothesis that the *b*_1_ coefficient is zero. In this way, we obtained *b*_1_ coefficient and its 95% confidence intervals for each plaque feature. A two-tailed *t*-test was used to examine differences between *b*_1_ coefficients from various plaque features.

1 $$\mathrm{HU }= {b}_{0 }+ {b}_{1}*\mathrm{log}\left(\mathrm{Energy}\right) + \left(1|\mathrm{ROI}\right) +\mathrm{ Error}$$

All statistical analyses were performed using R version 4.2.1 (R Foundation for Statistical Computing (Vienna, Austria)). The Benjamini–Hochberg procedure was applied to control for false discovery rate. We considered a Benjamini–Hochberg adjusted *p*-value of less than 0.05 as significant.

## Results

### Comparison of distinct plaque regions through the association between monoenergy reconstructions and measured HU

**Fig. 1:**
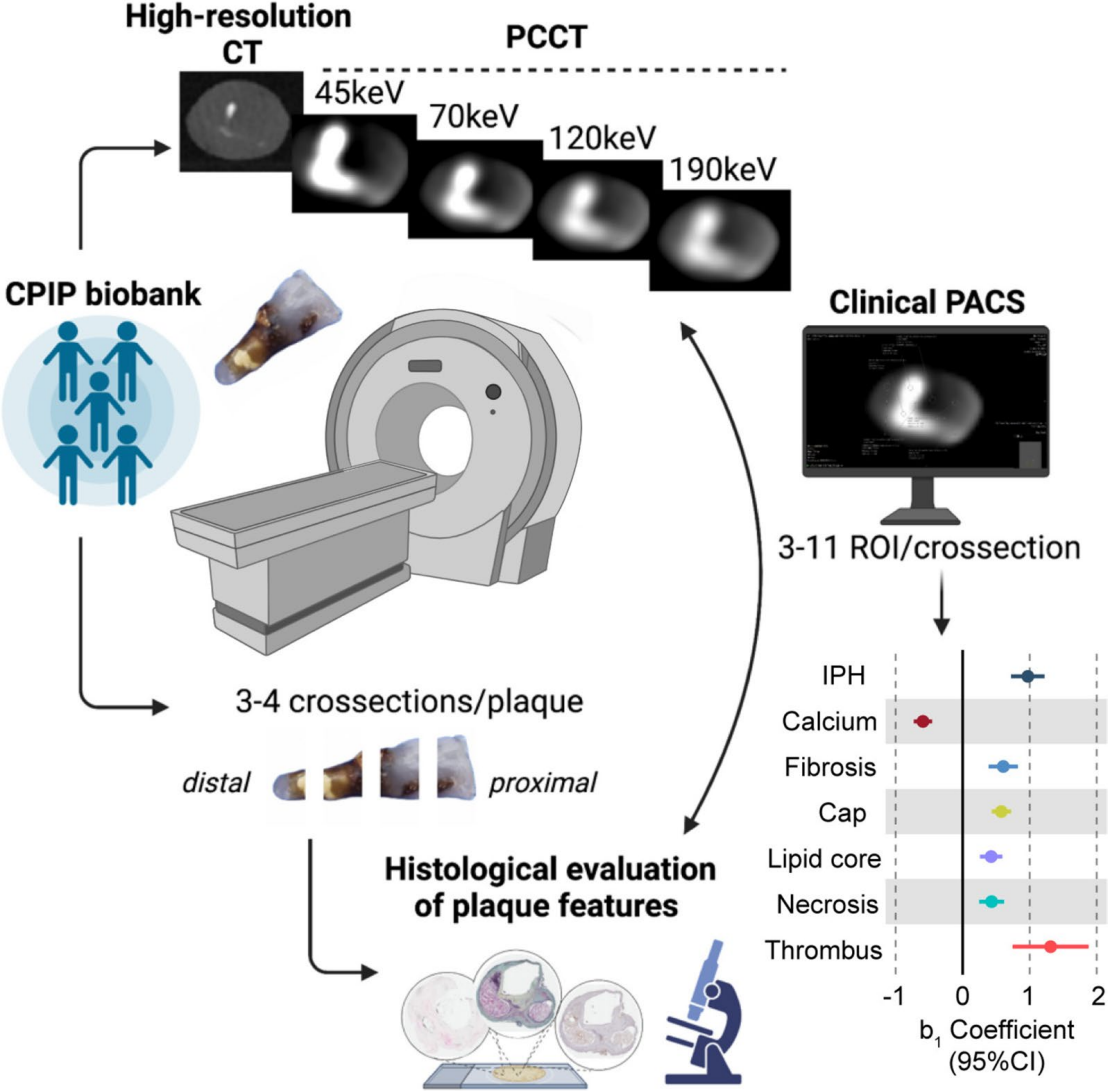
Atherosclerotic plaque features detected by PCCT. Evaluation of the histological characteristics of rupture-prone plaques that may be detected using PCCT showed this technique allows for the differentiation of well-known features of rupture-prone lesions beyond calcification, namely intraplaque haemorrhage and thrombus. Created in part with BioRender.com. *CPIP* Carotid Plaque Imaging Project, *CT* Computed tomography, *PACS* Picture Archiving and Communication System, *PCCT* Photon-counting CT, *ROI* Region of interest

**Fig. 2:**
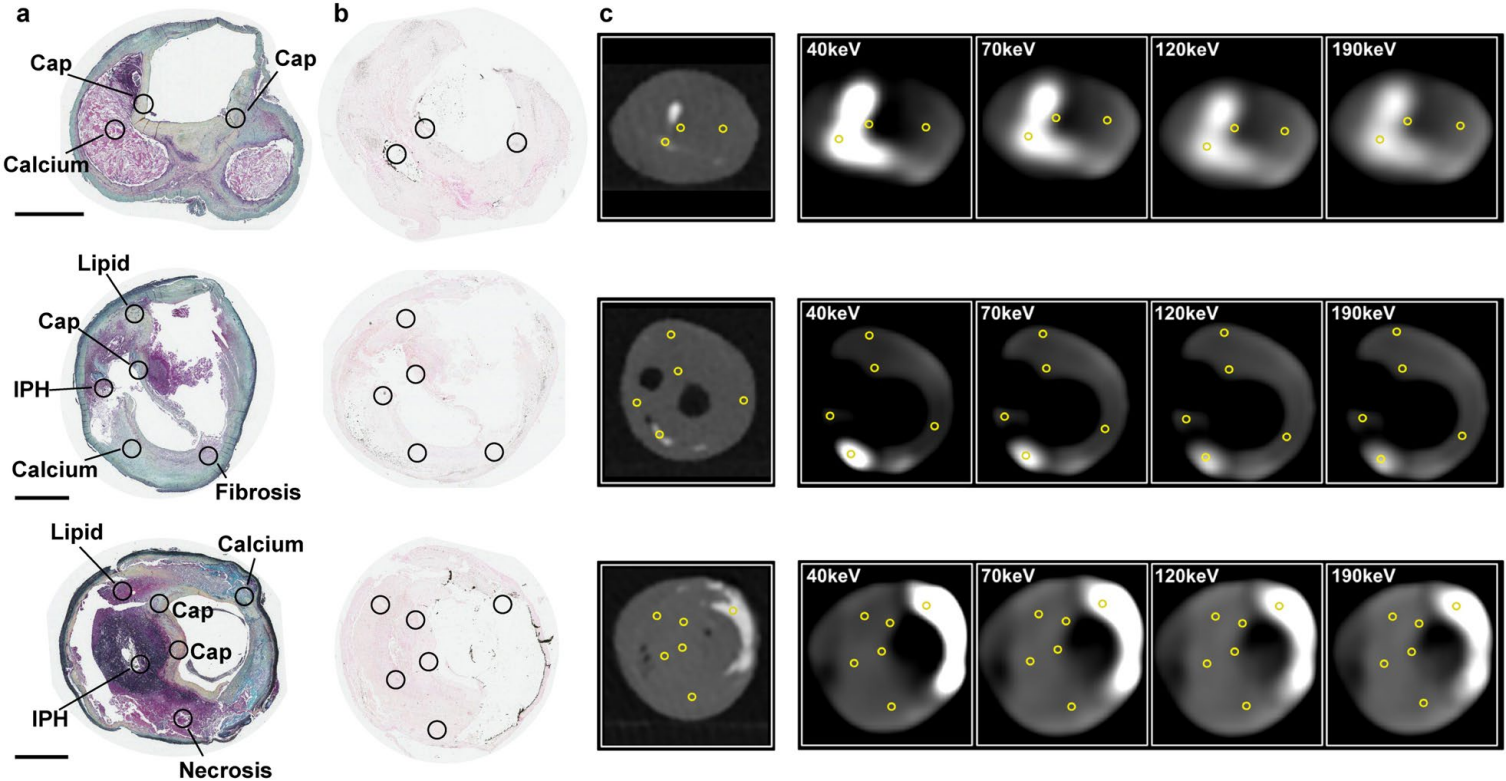
ROIs in histological and PCCT images. Representative plaque sections visualised with a Russel-Movat’s pentachrome stain (**a**) and von Kossa stain (**b**), with corresponding images of the same plaque locations (in the same plane) acquired during photon-counting CT (PCCT) scanning (**c**) are shown. Analysed plaque features—IPH, calcium, fibrous cap, fibrosis (elsewhere than in cap), lipid, necrosis and thrombus—are marked by circles and annotated. Black scale bars on the left represent 2 mm. *IPH* Intraplaque haemorrhage, *PCCT* Photon-counting computed tomography, *ROI* Region of interest

**Fig. 3:**
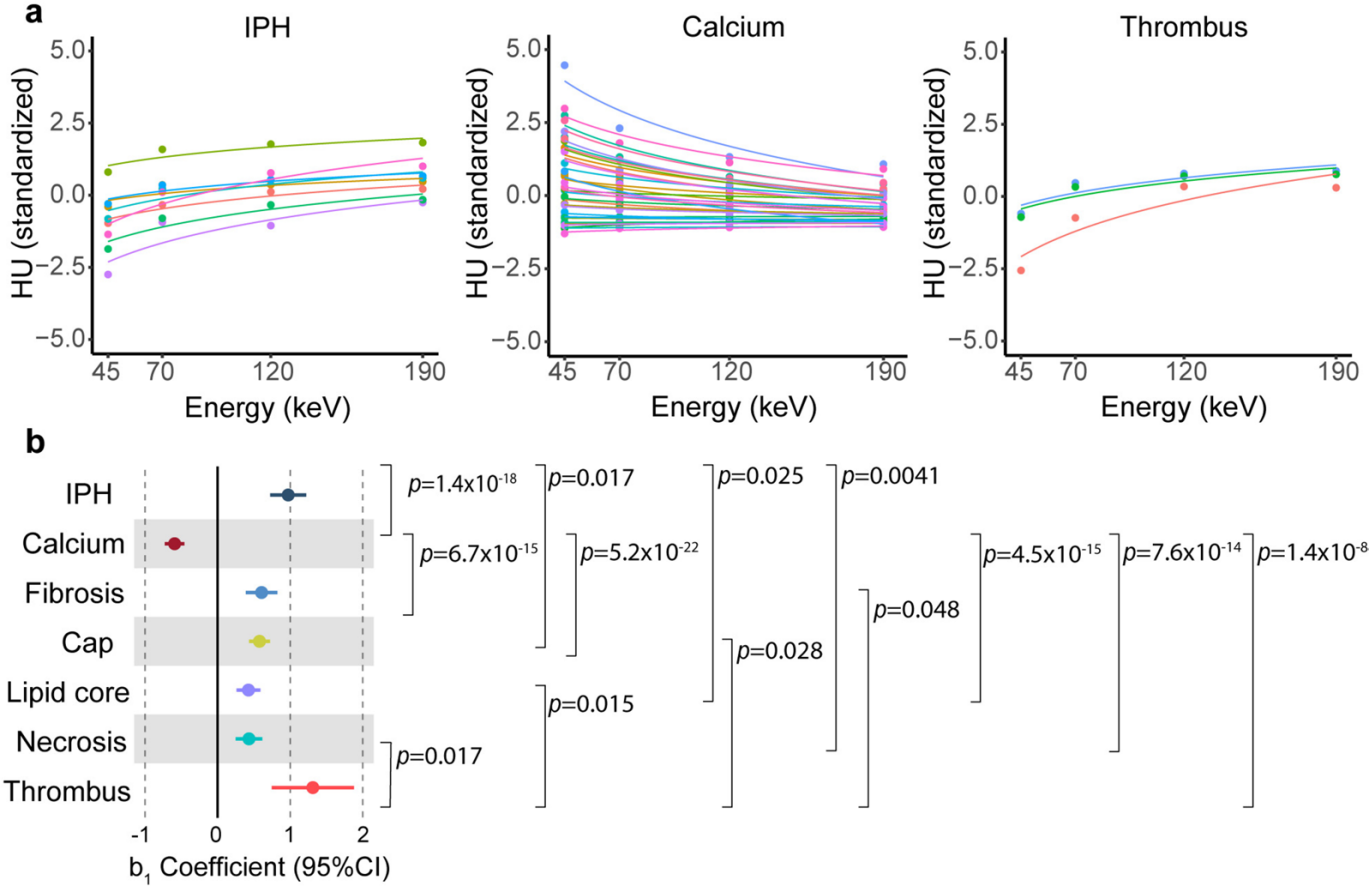
Density in HU of plaque features relevant for rupture-prone plaques. Coefficients with 95% confidence interval (CI) are shown for each analysed plaque feature, together with *p*-values signifying pair-wise comparisons of coefficients (**a**). The fitted lines visualise relationships between energy measurements (45, 70, 120 and 190 keV) and HU for all regions of interest representing intraplaque haemorrhage (IPH), calcium and thrombus (**b**). Differences in *b*_1_ coefficients from various plaque features were examined by *t*-test and multiple comparisons are corrected for using the Benjamini–Hochberg adjustment. IPH (*n* = 8), calcium (*n* = 34), fibrous cap (*n* = 25), fibrosis, elsewhere than in cap (*n* = 8), lipids (*n* = 8), necrosis (*n* = 5) and thrombus (*n* = 3)

Human advanced carotid atherosclerotic plaques were obtained at endarterectomies from five males with pre-operative cerebrovascular symptoms (Table 1). PCCT was performed *ex vivo* using clinical routine acquisition settings 120 kVp and 100 effective mAs (Fig. 1). ROIs were positioned on aligned high-resolution reference scan images and histologically stained sections denoting IPH, calcium, fibrosis, thrombus, necrosis and lipids: regions representing plaque features relevant to plaque rupture-risk according to previous studies [3, 9, 10] (Fig. 2 and Additional file 1: Fig. S1). The association between energy and HU for each plaque feature category was assessed by aggregating ROIs per category. The resulting energy-HU associations, presented as linear *b*_1_ coefficients, are shown in Fig. 3a, Additional file 1: Fig. S2 and Additional file 1: Table S1.

### PCCT can distinguish several rupture-prone plaque features

With the help of generating separate attenuation coefficients (*b*_1_) for each plaque feature of interest, each *b*_1_ coefficient was compared to assess whether the spectral behaviour of the respective feature categories differed.

These comparisons of plaque features through each respective *b*_1_ coefficient confirmed that calcium was distinguishable from all other analysed plaque features, *i.e.*, IPH (*b*_1diff_ = 1.57, 95% confidence interval [CI] 1.28–1.85, *p* = 1.410 × 10^-18^), fibrosis (*b*_1diff_ = -1.20, 95% CI -1.45 to -0.94, *p* = 6.705 × 10^-15^), fibrous cap (*b*_1diff_ = -1.17, 95% CI -1.37 to -0.97, *p* = 5.155 × 10^-22^), lipids (*b*_1diff_ = -1.02, 95% CI -1.23 to -0.80, *p* = 4.460 × 10^-15^), necrosis (*b*_1diff_ = -1.02, 95% CI -1.25 to -0.80, *p* = 7.586 × 10^-14^) and thrombus (*b*_1diff_ = -1.91, 95% CI -2.49 to -1.32, *p* = 1.436 × 10^-8^; Fig. 3b and Additional file 1: Table S2).

Several additional plaque features, well-known to be relevant for the risk of rupture, could be distinguished from each other. Significant differences were found for IPH compared to fibrous cap (*b*_1diff_ = 0.40, 95% CI 0.04–0.70, *p* = 0.017), to lipid (*b*_1diff_ = 0.55, 95% CI 0.25–0.85, *p* = 0.0025) and necrosis (*b*_1diff_ = 0.54, 95% CI 0.23–0.85, *p* = 0.004). Thrombus also differed significantly from fibrosis (*b*_1diff_ = -0.71, 95% CI -1.31 to -0.10, *p* = 0.048), fibrous cap (*b*_1diff_ = -0.74, 95% CI -1.30 to -0.17, *p* = 0.028), lipid (*b*_1diff_ = -0.89, 95% CI -1.48 to -0.30, *p* = 0.015) and necrosis (*b*_1diff_ = -0.88, 95% CI -1.48 to -0.28, *p* = 0.017; Fig. 3b and Additional file 1: Table S2).

## Discussion

In this study, we assessed which histologically verified features of rupture-prone plaques can be detected using PCCT, thus showing that the clinically used PCCT protocol enables the differentiation of well-known features of rupture-prone lesions beyond calcification, namely IPH and thrombus. This finding will be of high relevance to improve future diagnosis, risk stratification and atherosclerotic plaque monitoring through this novel technique, only recently made available for clinical use.

Currently, the clinical criteria for therapeutic interventions rely on the overall degree of stenosis and not on plaque features, even though many agree on the need for novel techniques to assess rupture-prone plaque characteristics to identify subjects at risk. In the coronaries, adverse plaque features, such as positive remodelling, napkin-ring sign, low attenuation and overall calcified plaque burden, have proved to predict cardiovascular events [11–13]. In the carotids, an association between the degree of stenosis and rupture-prone plaque features is not always found. Though the stenosis degree has been considered the major determining factor for carotid stroke incidents, recent evidence argues that specific alternate plaque features, including the presence of thrombus, may be more closely associated with the occurrence of ischemic stroke [14]. Furthermore, rupture-prone plaques are more common in asymptomatic patients, calling for their extended assessment beyond stenosis grade to improve risk stratification and optimise therapy [15].

With this study, we present qualitative proof-of-concept that PCCT, available to increasingly more departments, has great potential for use in plaque characterisation assessment. The next analytical step will be to develop a quantitative evaluation of plaques scanned *in vivo*. There is indeed a need for objective quantification of PCCT plaque images, an approach described by some groups such as Buckler et al. [16, 17] for CT angiography (CTA)—not PCCT—showing a high level of alignment between carotid plaque feature areas (of lipid-rich necrotic core, intraplaque haemorrhage and matrix tissue) as annotated on histopathological sections and by digital CTA analysis (using the software ElucidVivo; Elucid Bioimaging Inc., Boston, MA, USA). The same software has previously also been validated for CTA image analysis by comparison against molecular signatures determined by transcriptomics analysis, rather than histological annotation [18, 19].

Moreover, Min et al. [20] described quantitative atherosclerosis characterisation using an automated artificial intelligence-enabled web-based software platform (Cleerly Labs, Cleerly Inc, Denver, CO, USA), although measurements were performed for coronary arteries, and the output was primarily related to stenosis extent and volume. Analysing dual-energy-based CTA by semi-automated software (AutoPlaque research software, Cedars-Sinai Medical Center, Los Angeles, CA, USA) to quantify plaque characteristics, Hell et al. [21] found total, non-calcified and low-density coronary plaque volumes, as well as contrast density preference, to predict cardiac death in long-term follow-up in a large cohort of over 2,000 patients, while Ramanathan et al. [22] found the occurrence of carotid plaques to be underestimated by ultrasound compared to plaque assessment by CTA, with plaque composition determined by CTA, significantly different in individuals with and without carotid plaques by ultrasound.

With more similarity to our study, though using conventional dual-energy based CT techniques in five carotid endarterectomy specimens, Mannelli et al. [23] estimated accurate calcification sizes (as compared to histology), though with reduced sensitivity for smaller calcifications. This study further reports that calcification size measured on CT decreased systematically with increasing CT beam energy. Also using the dual-layer spectral detector CTA, Li et al. [24] distinguished carotid plaque morphological regions (compared to a histological reference) represented by attenuation spectrum curves across energy values. Analysing several components, the lipid-rich necrotic core was the main morphological feature differentiated from all other studied tissue categories (fibrous tissue, intraplaque haemorrhage and loose matrix). This contrasts with our study, principally discriminating IPH and thrombus from other plaque features. However, discrepancies are to be expected as experimental layouts differed significantly, importantly in tomography modes (dual-layer *CTA* compared to our PCCT), histological processing (haematoxylin/eosin overview stain only compared to our component-specific stains) and tissue state (an *in vivo* study compared to our *ex vivo* study).

PCCT is the latest clinically available technology in CT imaging. Compared to a conventional energy-integrated detector CT, PCCT uses a detector with semiconductors that directly converts x-ray photons to an electrical signal [25, 26]. This technique allows access to spectral information with high resolution, reduced electronic noise and increased contrast-to-noise [25, 26]. The use of spectral information from PCCT is a novel and robust strategy to obtain a more detailed plaque assessment. Interestingly, Dahal et al. [27] reported no significant differences between *ex vivo* spectral PCCT-derived radiological measurements *versus* histopathological measurements of fibrous cap thickness and area and lipid-rich necrotic core area. However, unlike ours, their equipment was not suitable for clinical application.

PCCT provides detailed plaque quantification. In the future, PCCT will potentially be able to detect photons in separated energy bins, which theoretically decreases energy overlap and further improves spectral separation. Another advantage of PCCT is that, at high resolution, it reduces the overestimation of calcium caused by blooming artefacts, which is common in conventional CT [28, 29].

Limitations of the current study are the limited number of carotid plaques included (5 plaques, though 97 ROIs) and that assessment was performed *ex vivo* and without contrast. The use of contrast agents would likely further improve the differentiations that we demonstrate here as pioneering proof of concept. Differences in contrast agent concentration and/or spectral attenuation may be detected, as described previously [30], even though only using a photon detector is not compatible with clinical applications. The use of CT with a multi-energy bin with tungsten as contrast media has indeed been suggested to improve carotid imaging in a pre-clinical setting with one sample [31]. However, we believe that distinguishing plaque features with PCCT in our study, even *ex vivo* and without contrast, is a clinically important advancement.

In conclusion, with this study, we propose the possibility of incorporating additional known features of rupture-prone plaques, such as IPH and the presence of thrombus [3, 9], into the evaluation of atherosclerotic plaque using PCCT. This is a potential breakthrough in cardiovascular imaging, which will ultimately improve diagnosis, risk stratification and monitoring of interventions by using plaque features as targets, beyond stenosis degree and calcification. Improved detection of rupture-prone plaques with PCCT will have the ultimate result of opening avenues to decrease global mortality and disability.

### Supplementary Information

**Additional file 1.** Supplementary Methods. **Supplementary Table S1.** Coefficients (b1 for log-transformed energy) of assessed plaque features. **Supplementary Table S2.** Difference in coefficients (b1 for log-transformed energy) for all comparisons of assessed plaque features. **Supplementary Figure S1.** Histologically stained plaque sections. **Supplementary Figure S2.** Hounsfield signatures of the additional plaque features (not in Figure 3) relevant for plaque vulnerability.

## Data Availability

The extent of sharing of datasets analysed/generated during this study containing deidentified participant data are subject to limitations specific to the ethical permit and general data protection regulation (GDPR, (EU) 2016/679). Because of the sensitive nature of the data collected for this study, requests to access the dataset from qualified researchers trained in human subject confidentiality protocols may be sent to the corresponding author.

## Abbreviations

CI: Confidence interval
CT: Computed tomography
CTA: CT angiography
IPH: Intraplaque haemorrhage
PCCT: Photon counting CT
ROI: Region of interest
