# Supplementary material for: Atherosclerotic plaque features relevant to rupture-risk detected by clinical photon-counting CT ex vivo: a proof-of-concept study
Source: Eur Radiol Exp. 2024 Jan 30;8:14. doi: 10.1186/s41747-023-00410-4 (PMC10825079; doi:10.1186/s41747-023-00410-4)
Supplement: Supplementary file 1 — Additional file 1. Supplementary Methods. Supplementary Table S1. Coefficients (b1 for log-transformed energy) of assessed plaque features. Supplementary Table S2. Difference in coefficients (b1 for log-transformed energy) for all comparisons of assessed plaque features. Supplementary Figure S1. Histologically stained plaque sections. Supplementary Figure S2. Hounsfield signatures of the additional plaque features (not in Figure 3) relevant for plaque vulnerability. [file 41747_2023_410_MOESM1_ESM.docx]

## **Atherosclerotic plaque features relevant to rupture-risk detected by clinical photon-counting CT *ex vivo* – a proof-of-concept study**

**ELECTRONIC SUPPLEMENTARY MATERIAL**

**Contents**

**Supplemental methods**

**Supplemental tables**

**Supplemental figures**

**Supplementary Methods**

**Study cohort: The Carotid Plaque Imaging Project (CPIP)**

The present study was approved by the Swedish Ethical Review Authority and conformed to the principles of the Declaration of Helsinki. All study subjects gave written informed consent and participation was not accompanied by any type of compensation.

Included in the study were five human carotid plaques collected during endarterectomies at the Vascular Department of Skåne University Hospital (Malmö, Sweden) from May 2019 to June 2020. The plaques included were randomly chosen from the CPIP biobank. Clinical characteristics of the included patients are presented in Table 1.

Briefly, indications for surgery were carotid artery stenosis >70% associated with ipsilateral symptoms (*amaurosis fugax*, transient ischemia attack or stroke). Symptoms were evaluated by a neurologist and the degree of stenosis obtained by flow velocities-based ultrasound [1].

Plaques were snap-frozen in liquid nitrogen immediately following excision at the operation room. Thereafter, plaques were thawed on ice, fixed in 4% formaldehyde, de-hydrated *en bloc* in a graded series of ethanol and embedded in paraffin.

**Histology**

Fibrosis was visualized through the standard Russel-Movat’s pentachrome stain. For the von Kossa calcium stain sections were fixed in 100% ethanol and then incubated in 1% silver nitrate, 5% sodium thiosulfate, and 0.1% fast nuclear red. Between incubations sections were rinsed in distilled H_2_O. Immunohistochemical staining was performed using primary antibodies against Glycophorin A (M0819; Agilent Technologies, Santa Clara, CA, USA) at 0.68µg/mL and oxidized low-density lipoprotein (oxLDL; ab14519, Abcam, Cambridge, UK) at 2 µg/mL. As negative controls, matched isotype controls were used at the same concentration as each primary antibody (ab18443 from Abcam and AB-105-C from R&D Systems for Glycophorin A and oxLDL, respectively). Prior to antibody incubations, sections were pretreated with sodium citrate antigen retrieval buffer (pH6) at 100°C for 15 min, with sections to be stained with the Glycophorin A antibody, additionally permeabilized with 0.5% Triton-X for 5 minutes. Endogenous peroxidase blocking was performed using 0.3% hydrogen peroxide (10 minutes), and protein block in 10% bovine serum albumin (30 minutes). Antibody incubation was followed by incubation with the MACH3 Mouse (for Glycophorin A) or Rabbit (for oxLDL) horseradish peroxidase (HRP)-Polymer Detection Kit according to the vendor’s instructions (M3M530H and M3R531H; Biocare Medical, Pacheco). Positive immunoreactivity was visualized using 3,3’diaminobenzidine (DAB; Vector Laboratories, Newark, CA, United States). Sections were counterstained with Mayer’s haematoxylin (Histolab Products AB, Askim, Sweden).

Stained slides were scanned and digitalized using a ScanScope digital slide scanner (Aperio Technologies Inc., Vista, CA, USA). Necrotic areas were defined as plaque regions devoid of cells or tissue.

**Reference for Supplementary methods**

1. Hansen F, Bergqvist D, Lindblad B, et al. (1996) Accuracy of duplex sonography before carotid endarterectomy--a comparison with angiography. Eur J Vasc Endovasc Surg.12(3):331-6.

**Supplementary Tables**

**Supplementary Table S1. Coefficients (b1 for log-transformed energy) of assessed plaque features.**

|  | b1 coefficient^[[1]](#footnote-1)^ | 95% Confidence Interval | | P-value^[[2]](#footnote-2)^ |
| --- | --- | --- | --- | --- |
|  |  | Lower | Upper |  |
| Intra-plaque haemorrhage | 0.97 | 0.72 | 1.22 | 2.413E-07 |
| Calcium | -0.59 | -0.73 | -0.46 | 8.970E-13 |
| Fibrosis | 0.61 | 0.39 | 0.82 | 2.962E-05 |
| Cap | 0.58 | 0.43 | 0.72 | 1.175E-10 |
| Lipid | 0.42 | 0.26 | 0.59 | 1.005E-04 |
| Necrosis | 0.43 | 0.25 | 0.62 | 5.558E-04 |
| Thrombus | 1.31 | 0.74 | 1.88 | 1.920E-03 |

**Supplementary Table S2. Difference in coefficients (b1 for log-transformed energy) for all comparisons of assessed plaque features.**

|  | **Difference in b1 coefficients** | **95% Confidence Interval** | | **P-value^[[3]](#footnote-3)^** |
| --- | --- | --- | --- | --- |
|  |  | **Lower** | **Upper** |  |
| IPH^[[4]](#footnote-4)^ versus (vs) calcium | 1.57 | 1.28 | 1.85 | 1.410E-18 |
| IPH vs fibrosis | 0.37 | 0.04 | 0.70 | 5.210E-02 |
| IPH vs cap | 0.40 | 0.11 | 0.68 | 1.752E-02 |
| IPH vs lipid | 0.55 | 0.25 | 0.85 | 2.549E-03 |
| IPH vs necrosis | 0.54 | 0.23 | 0.85 | 4.072E-03 |
| IPH vs thrombus | -0.34 | -0.96 | 0.28 | 3.228E-01 |
| Calcium vs fibrosis | -1.20 | -1.45 | -0.94 | 6.705E-15 |
| Calcium vs cap | -1.17 | -1.37 | -0.97 | 5.155E-22 |
| Calcium vs lipid | -1.02 | -1.23 | -0.80 | 4.460E-15 |
| Calcium vs necrosis | -1.02 | -1.25 | -0.80 | 7.586E-14 |
| Calcium vs thrombus | -1.91 | -2.49 | -1.32 | 1.436E-08 |
| Fibrosis vs cap | 0.03 | -0.23 | 0.29 | 8.709E-01 |
| Fibrosis vs lipid | 0.18 | -0.09 | 0.45 | 2.639E-01 |
| Fibrosis vs necrosis | 0.17 | -0.11 | 0.46 | 2.784E-01 |
| Fibrosis vs thrombus | -0.71 | -1.31 | -0.10 | 4.846E-02 |
| Cap vs lipid | 0.15 | -0.01 | 0.32 | 2.493E-01 |
| Cap vs necrosis | 0.15 | -0.04 | 0.33 | 2.639E-01 |
| Cap vs thrombus | -0.74 | -1.30 | -0.17 | 2.803E-02 |
| Lipid vs necrosis | -0.01 | -0.25 | 0.24 | 9.576E-01 |
| Lipid vs thrombus | -0.89 | -1.48 | -0.30 | 1.545E-02 |
| Necrosis vs thrombus | -0.88 | -1.48 | -0.28 | 1.664E-02 |

**Supplementary Figures**


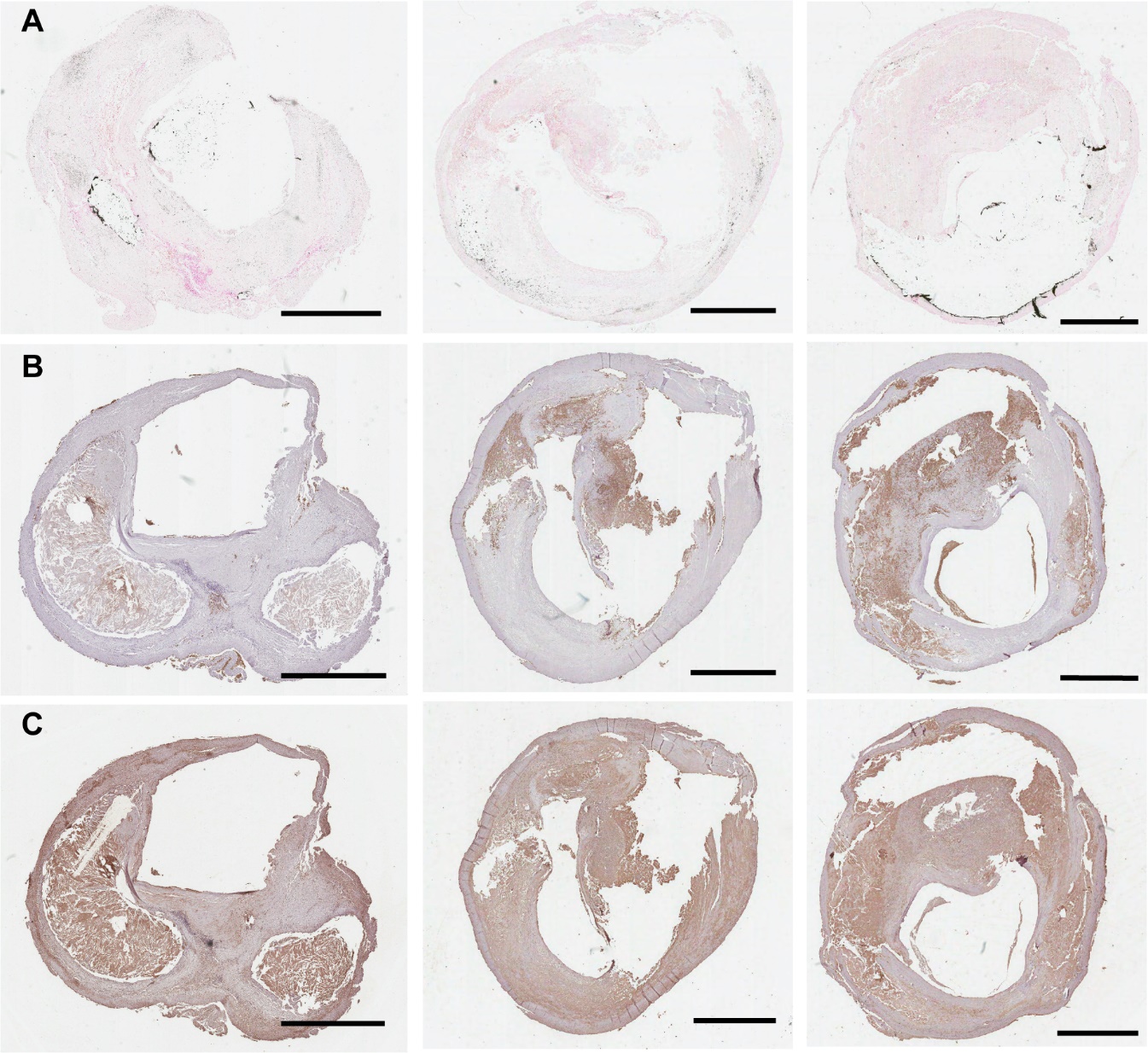


**Supplementary Figure S1. Histologically stained plaque sections.** A representative von Kossa stain for calcium-detection (**A**) and representative immunohistochemical detection of Glycophorin A (**B**) and oxidized low-density lipoprotein (oxLDL; **C**) are shown for plaque regions included in Figure 1.


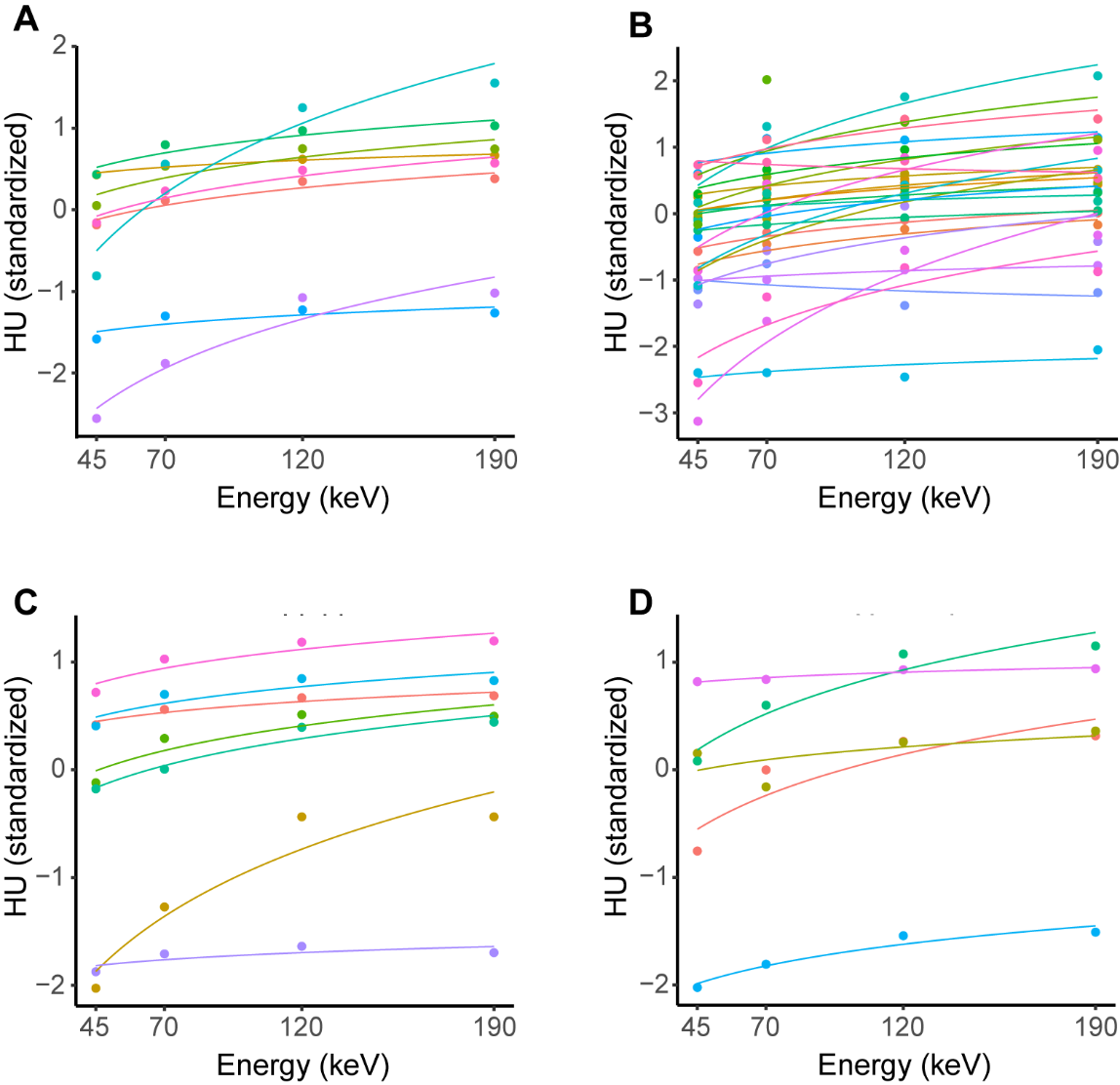


**Supplementary Figure S2. Hounsfield signatures of the additional plaque features (not in Figure 3) relevant for plaque vulnerability.** Energy measurements (45, 70, 120 and 190 keV) for all ROIs representing fibrosis (elsewhere than in the cap; **A**), fibrous cap (**B**), lipid (**C**) and necrosis (**D**) are visualized. N=8 for fibrosis, N=25 for cap, N=8 for lipid, N=5 for necrosis, N=15 for micro-calcifications, and N=19 for macro-calcifications.

1. b1 for log-transformed energy [↑](#footnote-ref-1)
2. Benjamini-Hochberg adjusted [↑](#footnote-ref-2)
3. Benjamini-Hochberg adjusted [↑](#footnote-ref-3)
4. Intra-plaque hemorrhage [↑](#footnote-ref-4)
